# Thinking about negative life events as a mediator between depression and fading affect bias

**DOI:** 10.1371/journal.pone.0211147

**Published:** 2019-01-25

**Authors:** Claire Marsh, Matthew D. Hammond, Matthew T. Crawford

**Affiliations:** School of Psychology, Victoria University of Wellington, Wellington, New Zealand; University of Pittsburgh, UNITED STATES

## Abstract

The current research examined the links between depressive symptomology and anxiety on the fading of affect associated with positive and negative autobiographical memories. Participants (N = 296) recalled and rated positive and negative events in terms of how pleasant or unpleasant they were at the time they occurred and at the time of event recollection. Multilevel mediation analyses identified evidence that higher levels of depressive symptoms were directly associated with lower affect fade for both negative and positive memories. Tests of indirect effects indicated that depressive symptoms were indirectly related to lower affect fade for negative (but not positive) autobiographical memories via the heightened tendency to think about negative (but not positive) memories. Anxiety was unrelated to affect fade both directly and indirectly. These results suggest that people higher in depressive symptoms retain more negative affect due to an increased likelihood of thinking about negative autobiographical events.

## Introduction

Coping with negative memories and savouring positive ones is thought to exemplify healthy emotion regulation [1–3]. Fading affect bias (FAB) is the well-established finding that affect associated with negative autobiographical memories fades faster than affect associated with positive autobiographical memories [3–5]. A decrease in the efficacy of this adaptive mechanism may contribute to the maintenance of depression or anxiety [6, 7] yet there has been little research elucidating the connection between depression, anxiety, and FAB. The studies that have done so [6, 7] have used a limited range of depression and anxiety symptomology inventory scores and have not examined these in concert. The current research uses a multilevel modelling technique to assess dimensional variation in depressive symptoms and anxiety to comprehensively examine the link between these psychopathologies and decreased affect fade. To do so, we report on a large sample with a much greater range of clinically-relevant depressive symptomology and anxiety than have been examined previously. Additionally, the current research expands on this by examining potential mediators based on frequency of thinking about, talking about, or writing about positive and negative autobiographical memories.

### Effects of depression on memory

Depression has been repeatedly shown to disrupt normal memory processes, such that people with depression often have overgeneral memory [8]. For example, Romero, Vazquez and Sanchez [9] found that participants classed as dysphoric recalled fewer specific memories and more general memories than those classed as non-dysphoric. People in the dysphoric category recalled the same number of positive and negative memories, whereas people in the non-dysphoric category recalled more positive memories. Kizilbash, Vanderploeg and Curtiss [10] studied the impact of depression, anxiety and co-morbid depression and anxiety on memory. Depression was associated with deficits in immediate recall and the total amount of new information acquired but retrieval and retention were unaffected. When depression and anxiety both occurred at higher levels, however, the adverse effects of depression occurred as before, but the retrieval of new information was also now impaired. Thus, a body of evidence suggests that depression has a variety of negative effects on memory.

In Walker et al.'s [6] study on dysphoria and fading affect bias, participants were asked to recall six emotionally intense memories and rate affect experienced both at event occurrence and upon recalling the event. In the first experiment participants were divided into either “dysphoric” or “nondysphoric” (BDI-I score of ≤ 9 classed as nondysphoric, *N* = 46; BDI-I score of ≥ 10 classed as dysphoric, *N* = 19; [11]). A significant FAB was evident in non-dysphoric participants, but no such pattern was found for the dysphoric group as affect associated with positive and negative events faded equally. In the second experiment a larger sample size was used (three separate replications) and individuals' BDI-I scores were used to divide them into categories based on the severity of their dysphoria using an approximate quintile split: low nondysphoric (BDI-I score 0–2, *N* = 69), moderate nondysphoric (BDI-I score 3–4, *N* = 62), high nondysphoric (BDI-I score 5–7, *N* = 67), marginally dysphoric (BDI-I score 8–12, *N* = 72) and dysphoric (BDI-I score 13+, *N* = 67). The goal of this division was to allow the researchers to assess changes in FAB as dysphoria increased and to test the level at which the effect reduced to nonsignificant. Evidence for FAB was found in the four lowest categories but was not evinced for those classed as dysphoric. Although significant FAB was found in the dysphoric group in some of the replications, the general pattern of findings suggested that for people with marginal dysphoria and dysphoria (as indicated by the divisions used by Walker and colleagues), negative fade decreased, and positive fade increased. The authors suggested that a BDI-I score of 10 or above was about the point at which FAB began to be affected. One limitation of the study is that because so few people were scored as moderately depressed, and no one was considered to be severely depressed (according to the BDI-I), the results cannot attest to how FAB operates across a wider range of depression scores, especially scores indicating clinically significant depression.

### Effects of anxiety on memory

Similar to depression it has been established that anxiety has a detrimental impact on memory function and can bias event recall [12, 13]. For example, Krans et al. [13] found that when asked to recall five *self-defining* autobiographical memories, people high in social anxiety were more likely to recall negative memories, especially events related to social anxiety, than did participants low in social anxiety. Glazier and Alden [14] found that even though people high in social anxiety accurately recalled the content and valence of positive and negative feedback immediately after the fact, they were impaired in recognizing feedback valence after a delay of one week. The authors suggest that over time, social anxiety may cause positive memories to be recalled as more negative which may explain why they do not receive as much psychological benefit from positive experiences as people low in social anxiety. Ricarte et al. [15] found that anxiety was indirectly associated with reduced memory specificity via brooding rumination, a finding replicated by Hallford and Mellor [12]. Specifically, anxiety had an indirect effect on autobiographical memory via rumination, such that increased rumination reduced memory specificity. Thus, previous literature has outlined several ways in which anxiety can distort or change memory recall, therefore it is important to study whether clinical levels of anxiety disrupt the normal memory process of fading affect bias because this could explain deficits in healthy emotion regulation.

The only paper to look at how trait anxiety affects FAB was conducted by Walker et al. [7] and comprises three studies. In the first study anxiety resulted in decreased FAB for moderate and high, but not for low anxiety groups (as measured by the DASS–[16]). Additional studies replicated this pattern using the Beck Anxiety Inventory (BAI [17]) using both retrospective (Study 2) and diary-based (Study 3) designs. Similar to their work on FAB and depression [6], the authors again divided their samples into discrete categories that did not always match the diagnostic scores for the tests that were being used. Additionally, neither of these studies measured both depression and anxiety in the same sample. Because of the frequent co-morbidity of these disorders (i.e., greater than 50% of people with current depression also have a current co-morbid anxiety disorder) it would be hard to determine whether the anxiety effects demonstrated might be due to depression, vice-versa, or, indeed, the interaction of the two [18–20].

### The current research

In addition to examining depressive symptomology and anxiety with a larger sample and across a broader range of scores (especially in terms of clinically-relevant levels of symptomatology), the current research also expands our understanding of how these psychopathologies affect FAB by examining whether the amount of thinking, talking, or writing about event memories changed depending on a person’s level of depressive or anxiety-based symptomology and whether this might serve as a mediator in the relationship with affect fading.

Available data on the relationship between thinking and fade is mixed. Ritchie et al. [21] found that involuntary private rehearsal was related to increased fade, present-focused voluntary private rehearsal was related to a decrease in fading of negative affect (relationship between present-focused voluntary private rehearsal and positive affect was inconsistent) and increased past-focused voluntary private rehearsal resulted in more fading of negative affect and less fading of positive affect. The relationship between depression and ruminative thought, however, is very well-established [22, 23]. Similarly, people high in anxiety are also typically high in pathological worry [24], suggesting that people high in anxiety may be thinking (i.e. worrying) about their event memories more than people low in anxiety (also see [12]). In the current study, we examined the relationship between depressive symptomology and amount of time spent thinking about the *specific* event memories. It should be noted, however, that this is not the same as rumination (e.g., as measured by a trait ruminative tendencies scale). This was done because we are interested in how thought about the specific event relates to how affect associated with that event fades, rather than ruminative tendencies in general (which tend to be highly correlated with depressive symptomology measures).

In terms of talking about autobiographical events, Skowronski, Gibbons, Vogl, and Walker [25] showed that events associated with social disclosure showed greater FAB. Because people with depression and/or anxiety often have less social support they may have fewer opportunities to discuss events and thus fewer opportunities to facilitate adaptive fade [26–28]. Finally, although it has been established that writing can be beneficial for people with depression [29, 30] and anxiety [31, 32], it was unclear how often people would write about event memories of their own volition, and how this may vary depending on levels of depression. To that end, we also examined the effect of writing on fade. To our knowledge, no literature currently exists on how writing might affect FAB.

Based on previous findings in the literature, we expected to find evidence of FAB overall, but that this differential fading would be significantly weakened (or non-existent) for individuals with high levels of depressive symptomatology (as measured by the BDI) and higher levels of anxiety (as measured by the BAI) as compared to individuals low on these measures. Exploratory analyses were conducted to ascertain whether the relationship between depression, anxiety and FAB was significantly mediated by thinking, talking, or writing about event memories.

## Method

### Participants

A total of 302 (46 males, 256 females) Victoria University of Wellington students in a first year psychology course participated in the study and received credit toward the completion of the research participation coursework. The average age of participants was 19 years (*SD* = 3.15; range 17–45). Available data were 1208 positive memories and 1208 negative memories. From the initial 302 participants, six (< 2%) were removed for either not providing any memories or failing to follow instructions. This left 296 individuals (42 males, 254 females) and 1184 positive memories and 1184 negative memories. Ethical approval for this research was granted by the Victoria University of Wellington School of Psychology Human Ethics Committee.

### Memory-level measures (Level 1)

#### Event recollection

Events were recorded using a retrospective method [5]. Participants were asked to recall four positive and four negative memories, avoiding anything that they felt was too personal to share. The order in which participants completed the positive and negative events was counterbalanced across the study. For each event recording, participants rated the importance and vividness of that memory on a seven-point scale from 1 (not at all important/vivid) to 7 (very important/vivid). Participants also rated how often they had talked, written, and thought about each event memory on separate seven-point scales from 1 (not at all) to 7 (very often). In order to ensure that participants engaged with the event recollection task and that they provided enough information to indicate that they thought about the event in detail, participants were unable to advance until at least eight minutes had elapsed (i.e., four minute minimum for positive events and four minute minimum for negative events). Other than setting this minimum time, participants could write for as long as they wished. In fact, participants in this study spent, on average, about twenty-three minutes on the autobiographical recollection task (Negative events M = 11.49, SD = 4.75; Positive events M = 11.08, SD = 4.06).

#### Fading affect bias

For each memory, participants made two ratings of the event on a scale ranging from -3 (extremely unpleasant) to +3 (extremely pleasant) both for how pleasant/unpleasant the event was *at the time that it happened* and also *right now*. Affect fade, then was calculated as the difference between (un)pleasantness at the time of the event and at the time of retrieval. For example, if a negative event was rated as a negative three at the time of the event, and a zero at recollection, this represents a fade of three points (i.e., negative events fade by becoming more positive); a positive event originally rated a three, but at recollection a zero, likewise represents a fade of three points (i.e., positive events fade by becoming less positive).

A potential criticism of this method involves the retrospective nature of the event rating; specifically, that relying on individuals to accurately recall how they felt about a particular event—at the time that it occurred—may introduce memory bias. Although a diary-based study would appear to be a stronger method for dealing with the potential for biased affect recollection, it should be noted that researchers have demonstrated FAB using a variety of approaches, including experiments, diary studies, and retrospective studies across multiple cultures [5, 21, 33, 34]. The overall consistency of these different approaches indicates that FAB is a robust and replicable phenomenon that emerges under a variety of study conditions, and that retrospective bias is not responsible for FAB findings.

### Participant-level measures (Level 2)

#### Beck Depression Inventory (BDI)

The BDI-I is a 21 item scale that measures depressive symptomology over the previous two weeks [11]. For each item, participants pick one out of four statements that best describes their mood in the last two weeks. An example item is "I do not feel sad; I feel sad; I am sad all the time and cannot snap out of it; I am so sad and unhappy that I cannot stand it". Potential scale scores range from 1–63 with higher scores indicating increased depressive symptomology. Scores are sometimes divided into five categories; normal (1–10), mild mood disturbance (11–16), borderline clinical depression (17–20), moderate depression (21–30) and severe & extreme depression (31–40 & over 40 respectively). We kept the BDI as a continuous measure in the following analyses to retain statistical power.

#### Beck Anxiety Inventory (BAI)

The BAI is a 21 item scale that measures symptoms of anxiety experienced in the past month [17]. For each item participants rate how much each symptom (e.g., “Unable to relax”) bothered them on a four option scale with endpoints 0 = not at all and 3 = severely. Scores range from 0–63 with higher values indicating increased anxiety. Traditionally, scores of 0–7 represent minimal anxiety, 8–15 mild anxiety, 16–25 moderate anxiety, and 26–63 indicate severe anxiety. As with the BDI, we modelled this measure as a continuous variable in our analyses.

### Procedure

Participants completed the study at a time and place of their choosing as all of the instructions and measures were presented online using Qualtrics Survey Software [35]. Prior to beginning the study, participants were cautioned that it would take approximately 30 minutes to complete the survey and that it must be completed in one sitting. After reading the information sheet and consenting to participate, respondents completed all of the measures as described above. Positive and negative event recollection always occurred together (with order counterbalanced), and measures of depression and anxiety always occurred together (again, with order counterbalanced). Additionally, the order in which participants completed either the event recall or the clinical measures was counterbalanced so that half of the sample completed the event recollection tasks prior to completing the other measures; the other half completed the clinical measures prior to engaging in the event recall task. Across the four counterbalancing conditions, each task appeared in each position an equal number of times and no order effects were observed. Upon completion of all measures, participants were debriefed via the survey platform and thanked for their participation.

### Statistical analysis—Multilevel modelling

Data were structured so that participants’ memories of events (level one; *N* = 2368) were nested within participants (level 2; *N* = 296). The following analyses were maximum likelihood, robust standard error (MLR), multilevel models conducted in MPlus 7.2 [36]. Multilevel modelling was necessary to account for the inherent dependence between the affect of memories reported by each participant [36, 37]. This approach is superior to regression modelling because we can examine within-person effects (i.e., associations with memory valence) and between-person effects (e.g., associations with depressive symptoms) simultaneously. Intercepts were modelled as random (i.e., allowed to vary across participants) and predictor variables were grand-mean centered.

As an example equation for level 1 of this multilevel model is displayed in Eq 1.

$$F_{mp}=b_{0p}+b_{1p}\;(valence\;for\;memory\;m)+e_{mp}$$ (1)

In this equation, affect fade (F) for person *p* for a particular memory *m* is a function of an intercept (b_0_ for person *p*), the valence for that memory (b_1_) which tests the presence of the fading affect bias, and an error term (*e*_mp_). The main effect of memory valence represents the magnitude of the fading affect bias. Any interaction terms in the following models that included memory valence allowed us to test whether the model parameters (e.g., the link between writing about a memory and affect fade for that memory) statistically differed between positive and negative memories. The following equations (at level 2) tested our central research questions.

$$b_{0p}=B_{00}+B_{01}\;(depression\;score\;for\;person\;p)+u_{0j}$$ (2)

$$b_{1p}=B_{10}+B_{11}\;(depression\;score\;for\;person\;p)+u_{0j}$$ (3)

The term *B*_01_ in Eq 2 represents the extent to which depression was associated with affect fade for memories, testing whether people higher in depression experienced more vs. less affect fade for memories in general, with an accompanying error term (*u*_0j_). Simultaneously, the term *B*_11_ in Eq 2 represents the extent to which depression moderated the Level 1 slope of affect fade on memory valence (see Eq 1). This moderating effect represents the extent to which the fading affect bias was greater (or diminished) for people higher (vs. lower) in depressive symptoms.

## Results

The sample mean score on the BDI was 12.01 (SD = 9.75), and for the BAI was 18.81 (SD = 13.17). Table 1 presents the number of participants who fell within each level of the diagnostic categories for these measures. Unsurprisingly, scores on the BDI and the BAI showed a reliable positive correlation (*r*(294) = .60, *p* < .001). Age did not significantly correlate with either scores on the BDI (*r*(294) = .04) or the BAI (*r*(294) = .03).

**Table 1:** Frequency table of participants scoring within each clinical diagnostic level of the BDI and BAI.

		N	Sample %	Cumulative %
BDI Categories				
	Normal	163	55.1	55.1
	Mild Mood Disturbance	52	17.6	72.6
	Borderline Clinical Depression	23	7.8	80.4
	Moderate Depression	41	13.9	94.3
	Severe & Extreme Depression	17	5.7	100.0
BAI Categories				
	Minimal	69	23.3	23.3
	Mild	71	24.0	47.3
	Moderate	67	22.6	69.9
	Severe	89	30.1	100.0

### Fading affect bias

In a preliminary model, the sample was tested for the presence of FAB by regressing level of affect fade on memory valence. Overall, and as expected, the affect associated with positive events faded less than the affect associated with negative events (*B* = -.680 [95% CI = -.743 to -.617], *t* = -21.193, *p* < .001).

#### Testing a direct link between BDI scores and affect fade

The first test was conducted to assess the hypothesis that people with higher BDI scores would have a smaller fading affect bias compared to people with lower BDI scores.

Results again identified the fading affect bias as indicated by the main effect of memory valence (*B* = -.680 [95% CI = -.743 to -.617], *t* = -21.247, *p* < .001). Results also indicated that higher BDI scores were significantly associated with lower affect fade in general (*B* = -.010 [95% CI = -.016 to -.004], *t* = -3.163, *p* = .002). Interestingly, no significant moderating effect of depression emerged on the link between memory valence and affect fade (*B* = .001 [95% CI = -.006 to .008], *t* = 0.356, *p* = .722). This finding indicated that people with higher depressive symptoms retained relatively more affect of memories regardless of memory valence, but did not differ in the extent to which they exhibited the fading affect bias compared to people with lower levels of depressive symptoms.

#### Testing a direct link between BAI scores and affect fade

Walker et al. [7] provided evidence that the fading affect bias was less pronounced in high-anxiety groups. Our hypothesis that Walker et al.’s finding would be replicated was evaluated by conducting models (equivalent to those described above) to test whether anxiety symptoms predicted lower affect fade. Contrary to prior evidence, no significant direct link emerged between anxiety symptoms and affect fade for memories (*B* = -.003 [95% CI = -.007 to .001], *t* = -1.336, *p* = .182), regardless of whether the memories were positive or negative (*B* = .001 [95% CI = -.004 to .006], *t* = 0.367, *p* = .713).

#### Testing for the effect of comorbid depression and anxiety

Subsequently, an identical model to the above was conducted including anxiety symptoms and depressive symptoms, and an interaction term between anxiety symptoms and depressive symptoms, as simultaneous predictors of affect fade. In this model, depressive symptoms remained predictive of lower affect fade (*B* = -.092 [95% CI = -.170 to -.014], *t* = -2.317, *p* = .021), and anxiety symptoms remained unrelated to affect fade (*B* = .027 [95% CI = -.024 to .077], *t* = 1.023, *p* = .306). A significant interaction emerged between anxiety symptoms and depressive symptoms predicting affect fade (*B* = -.012 [95% CI = -.019 to -.005], *t* = -3.150, *p* = .002), that did not differ between positive and negative memories (*B* = .011 [95% CI = -.036 to .058], *t* = 0.446, *p* = .655). Simple slopes of this interaction indicated that depressive symptoms were related to reduced affect fade when people were higher in anxiety symptoms (1 SD above the mean; *slope* = .145, [95% CI = -.224 to -.067], *t* = -3.150, *p* < .001) relative to when people were lower in anxiety symptoms (1 SD below the mean; *slope* = -.039 [95% CI = -.137 to .059], *t* = -0.776, *p* = .437). However, it should be noted that slope estimates for a person high in depressive symptoms but low in anxiety symptoms (or vice versa) are relatively less conclusive because of the high proportion of comorbidity in the sample (see Table 2). Taken altogether, the patterns of tests including anxiety symptoms indicated that they did not account for the association between depressive symptoms and reduced affect fade.

**Table 2:** Frequency table of co-morbidity.

BDI Score	BAI Score			
	Minimal	Mild	Moderate	Severe
Normal	59 (19.9%)	49 (16.6%)	30 (10.1%)	25 (8.5%)
Mild Mood Disturbance	6 (2.0%)	13 (4.4%)	19 (6.4%)	14 (4.7%)
Borderline Clinical Depression	1 (0.3%)	2 (0.7%)	9 (3.0%)	11 (3.7%)
Moderate Depression	3 (1.0%)	6 (2.0%)	6 (2.0%)	26 (8.9%)
Severe and Extreme Depression	0 (0.0%)	1 (0.3%)	3 (1.0%)	13 (4.4%)

#### Testing indirect links between BDI scores and affect fade

**Fig 1:**
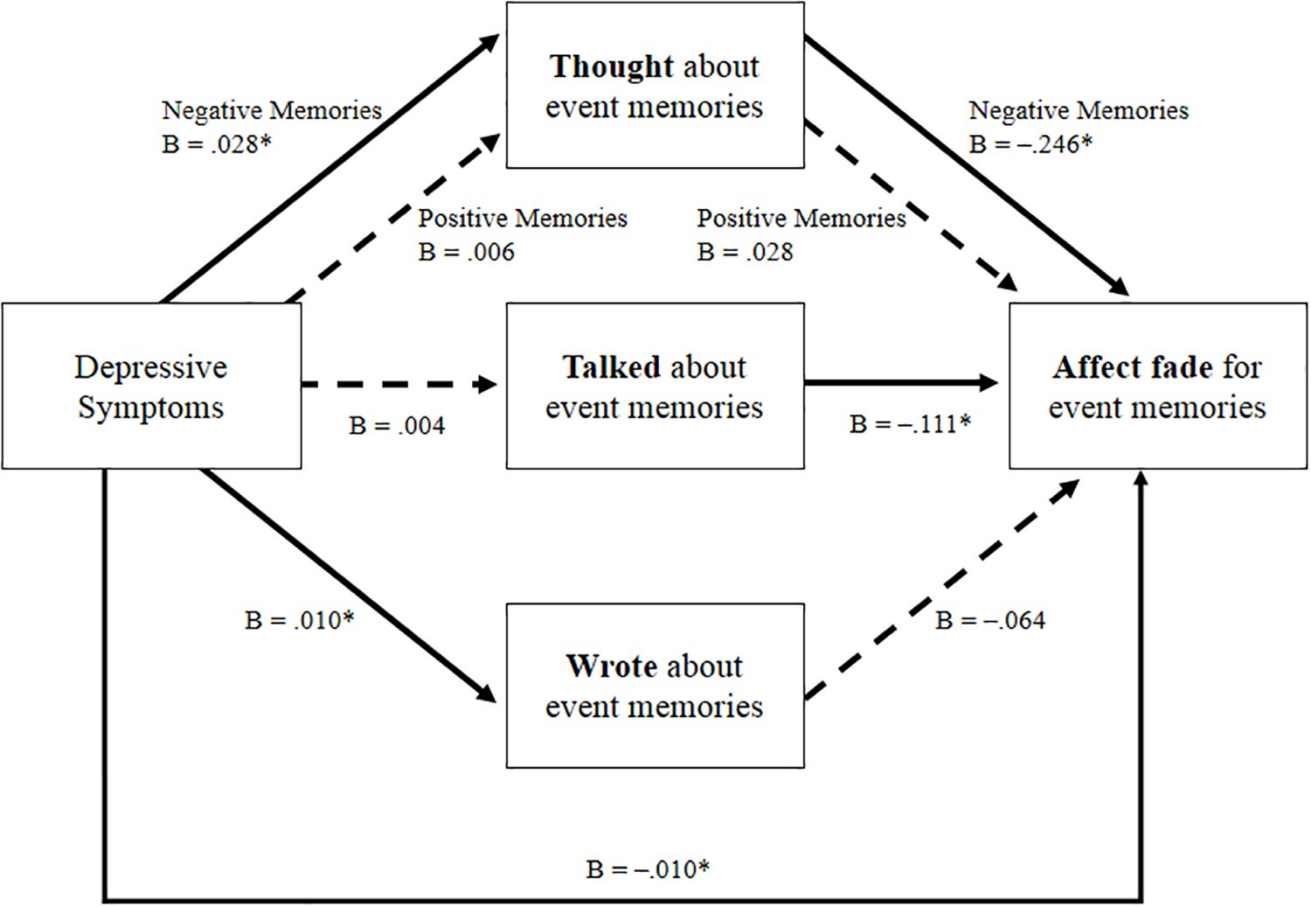
Multilevel structural equation model of the links between depressive symptoms and affect fade, mediated by the extent to which people thought, talked about, and wrote about those memories. Model parameters for memories of positive and negative events are pooled unless significant interactions by memory valence emerged, in which case we display the conditional effects (i.e., simple slopes). Dashed lines indicate non-significant parameters. *p < .05. Fit statistics indicated acceptable fit for the data (RMSEA = .033; SRMR *Level 1* = .017; SRMR *Level 2* = .008; CFI = .98). The chi-square test of model fit was significant (χ^2^ = .32,66, *p <* .001), which was expected given the large sample size.

Our first model indicated that people higher in depressive symptoms experienced lower affect fade for both positive memories and negative memories compared to people lower in depressive symptoms. Our next models assessed the exploratory hypothesis regarding potential reasons for this link. A multilevel structural equation model was again used, in which depressive symptoms (Level 2) predicted the extent to which people (1) thought about, (2) talked about, and (3) wrote about their memories (Level 1 variables), and in turn, predicted affect fade (see Fig 1; Full results reported below). As in our prior analyses, memory valence (positive vs. negative) was included as a predictor to assess fading affect bias. In addition, we included interaction terms between memory valence and thinking, talking, and writing about memories to assess the extent to which effects of these potential mediators differed between positive and negative memories.

In this model, significant effects indicated that people with higher BDI scores both thought about (*B* = .017 [95% CI = .007 to .026], *t* = 3.506, *p* < .001) and wrote about their memories (*B* = .010 [95% CI = .004 to .016], *t* = 3.033, *p* = .002) more than people with lower BDI scores. There were no significant differences in talking about memories across different levels of BDI scores (*B* = .004 [95% CI = -.004 to .013], *t* = 1.035, *p* = .300). These effects were qualified by one significant interaction between BDI scores and valence of memories predicting the extent to which people thought about memories (*B* = -.011 [95% CI = -.017 to -.005], *t* = -3.360, *p* < .001). Estimation of the simple slopes indicated that higher BDI scores were related to thinking more about negative memories (*B* = .028 [95% CI = .016 to .040], *t* = 4.619, *p* < .001) but were not significantly related to thinking about positive memories (*B* = .006 [95% CI = -.005 to .016], *t* = 1.049, *p* = .294). These results suggested that people with higher BDI scores tended to engage more in two forms of memory-related behaviors—thinking about negative events and writing about their memories.

The next model examined the extent to which of the potential mediators—thinking about memories, talking about memories, and writing about memories—predicted affect fade. Two potential mediators were ruled out. First, depressive symptoms were not related to differences in talking about memories (B = -.003 [95% CI = -.007 to .001], *t* = -1.703, *p* = .088). However, there was a significant positive association between talking about a particular memory and affect fade for that memory (*B* = .111 [95% CI = .043 to .179], *t* = 3.181, *p* = .001). This association did not significantly differ between positive and negative memories (*B* = -.061 [95% CI = -.123 to .001], *t* = -1.923, *p* = .055). Second, depressive symptoms were related to writing about memories more. However, writing about memories was not significantly associated with affect fade for those memories (*B* = .064 [95% CI = -.013 to .141], *t* = 1.620, *p* = .105), regardless of whether memories were of positive events or negative events (*B* = .029 [95% CI = -.040 to .098], *t* = 0.820, *p* = .412). Thus, neither talking about memories nor writing about memories were potential mediating variables.

Finally, it was expected that increased thinking about event memories would be related to lower affect fade. As predicted, the more that people thought about a particular memory, the lower the affect fade for that memory (*B* = -.109 [95% CI = -.161 to -.056], *t* = -4.069, *p* < .001). This association was qualified by a significant interaction with memory valence (*B* = .137 [95% CI = .083 to .191], *t* = 4.940, *p* < .001). Tests of simple slopes revealed that increased thinking about memories was related to decreased affect fade for memories of negative events (*B* = -.246 [95% CI = -.319 to -.173], *t* = -6.588, *p* < .001) but not for memories of positive events (*B* = .028 [95% CI = -.050 to .106], *t* = 0.708, *p* = .479). Even when accounting for the additional variables in the model, there was still a significant direct link between depressive symptoms and lower affect fade (*B* = -.010 [95% CI = -.017 to -.004], *t* = -3.027, *p* = .002). Thus, thinking more about negative memories was linked with lower affect fade and therefore a potential partial mediator of the link between depressive symptoms and lower affect fade.

**Indirect Effects**: Finally, following guidelines by Hayes [38], we estimated the indirect effect between depressive symptoms and lower affect fade for memories, via greater thinking about those memories. We simultaneously estimated indirect effects for positive memories and for negative memories because the analyses reported above illustrated that the parameters in this pathway were moderated by memory valence. Results indicated a significant indirect effect for the pathway “depressive symptoms → thinking about memories → affect fade” for negative memories (*indirect effect* = -.007 [95% CI = -.010 to -.003], *t* = -3.854, *p* < .001). In contrast, there was no indirect effect for the pathway “depressive symptoms → thinking about memories → affect fade” for positive memories (*indirect effect* = .000 [95% CI = .000 to .001], *t* = 0.580, *p* = .562). It is reasonable to assume that one consequence of thinking about an event would be the maintenance of the affect associated with that event. That is, thinking about positive events should better maintain the positive affect associated with the memory (sometimes referred to as *savoring*) and thinking about negative events should maintain the negative affect associated with that memory. In other words, thinking about events should decrease the amount of affect fade seen. The fact that we see differential affect maintenance, with negative affect fading less (compared to positive affect) with increased thinking, has not—to our knowledge—been demonstrated previously. Because depression is highly correlated with both ruminative thinking and general emotion regulation dysfluencies, picking apart the exact nature of this effect is beyond the scope of the current study. However, these results do suggest that the association between people’s depressive symptoms and lower affect fade for negative memories was partially due to their increased thinking about negative memories.

## General discussion

Our memories of the past inform our current world view and shape our daily behaviour. It is vital we have a clear understanding of how memory processes work, and what may help or hinder them, because autobiographical memory is essential to the construction of the self. The purpose of this research was to investigate the manner in which clinically significant levels of depressive symptomatology and anxiety impact FAB.

Overall, evidence for FAB was found in the current study. That is, the affect associated with negative events faded more than the affect associated with positive events. However, as depressive symptomology increased, this differential fading of affect did not occur. These results expand on those reported by Walker at al. [6] by demonstrating this relationship with a much larger and significantly more diverse (i.e., in terms of BDI range) sample of participants. In that earlier study, the authors were unable to investigate how FAB was affected by clinically-relevant levels of depressive symptomatology due to having so few participants classed as having “moderately” high levels and no participants classed as having “severe” levels [11]. In contrast, the present study included 13.9% of participants who were considered as showing moderate levels of depressive symptomology (based on the BDI-I categories) and 5.7% would be classified as showing severe or extreme levels. These numbers are substantial thus offer stronger evidence about how clinically-relevant levels depressive symptomology affects affect fade.

Additionally, by modelling depression as a continuous variable, rather than dichotomizing depression as in prior research [6], the current study was able to retain more information from the measures and provide a more sensitive analysis of this relationship [39]. This analytic approach allowed us to examine what happened to FAB as symptomology related to depression increased: FAB was entirely absent for people high in depressive symptomology because those individuals experienced lower affect fade for both positive and negative memories (that is, no differential affect fade). Whereas Walker et al.’s [6] findings suggested that the decrease (or absence) of FAB was due to decreased fading of negative affect and increased fading of positive affect, the current study only offers support for decreased fading of negative affect.

The current study also suggests an explanation for the decrease in negative affect fade, which expands again on the work by Walker et al. [6]. Consistent with a large body of literature showing that rumination is strongly correlated with, and predictive of, depression [23, 40], the current study found that people with higher BDI scores thought more about their memories than people with lower BDI scores. This was especially true in terms of thinking about negative autobiographical events. That is, one of the things that contributes to the lack of negative affect fading is the fact that people high in depression thought about negative event memories more than people lower in depressive symptomology. The more individuals thought about these *specific* negative memories, the less the affect associated with them faded. Again, it should be noted that our measure of *thinking about* an event is not the same as rumination, per se. That is, ruminative thought refers to the tendency toward repetitive, and often unwanted, thinking about an event. Our measure of thinking does not differentiate between voluntary and involuntary (i.e., intrusive) thinking, nor whether the thought itself involved re-living the emotional content of the memory. However, given the pattern of results and the extant literature on depression and trait-level ruminative tendencies, it is reasonable to speculate that the type of thinking that individuals with higher-levels of depressive symptomology are engaging in probably fits under the general rubric of *ruminative thought*.

Surprisingly, and counter to predictions, anxiety was unrelated to the occurrence of FAB in the current study, thus diverging from the findings of Walker et al. [7]. In those earlier studies increased levels of trait anxiety led to a decrease in differential fading. One possibility for the failure to replicate those findings may have to do with the manner in which anxiety scores were treated in the earlier studies versus how they were used in the current work. That is, Walker and colleagues deviated from the usual (i.e., diagnostic) categories of both the DASS and the BAI in order to create categories that had relatively even numbers of participants thus were more suitable to their statistical analyses. In the first study Walker et al. created a low anxiety category based on DASS scores of only 0–3, and a moderate anxiety category based on DASS scores of 4–9 whereas in reality, DASS scores up to 7 are considered normal. In the second study the authors also manipulated their categories by shifting the boundaries down, so that people classed on the BAI as having minimal anxiety were included in Walker et al.’s moderate anxiety group and people classed on the BAI as mildly anxious were included in the high anxiety group.

Walker et al. suggest (citing [41]) that because, in the clinical area of psychology in particular, continuous measures are necessarily divided into discrete categories they are justified in using categories in their analyses. However, the category boundaries used did not match those used diagnostically by clinical practitioners. Furthermore, DeCoster et al. [41] state that the argument about retaining categories because measures in clinical psychology necessarily have categories is relevant if the research is checking how well the measure works in a real-world setting. If the purpose of the research to ascertain the relationship between the construct underlying the measure and another variable, then analyses which preserve the continuous nature of the measurements are the best to use [42, 43]. The use of multi-level modelling provides a more sensitive test of the relationship between anxiety and affect fading than a more categorical analysis would allow.

Another potential limitation of the previous work was that depression and anxiety were not measured in the same samples which is a weakness given rates of co-morbidity [20, 44]. Furthermore, it had already been established that dysphoria (and therefore likely depression) diminished FAB [6]. Without controlling for the known disruptive effects of depression, it is not possible to determine whether the diminishing of FAB with increased anxiety is specific to anxiety alone. It is possible that the effects demonstrated in the Walker et al. [7] study may be due to level of depression or perhaps the interaction between depression and anxiety. It is not possible to know about rates of comorbidity in that work as measures of depression were not collected.

As a reference point, in the present study 16.2% of the total sample scored in the moderate or severe category for both the BAI and the BDI-I. This comprises 58 participants who scored either moderate or severe on the BDI-I, and 48 (82.7%) of those same participants also scored moderate or severe on the BAI indicating significant co-occurrence. Indeed, the only time in the present study that anxiety had a significant association with affect fade was when it co-occurs with depression. In this instance a larger decrease in fade was evinced compared to depression alone. This finding raises the possibility that reductions in affect fade attributed to anxiety in previous work may have been the effect of depression and anxiety combined, though this remains speculative.

### Limitations

Due to the correlational nature of the current study, and the fact that measures were collected in the same session, it is not possible to draw causal conclusions regarding direction within the mediation model. That is, to offer evidence for the direction of the model, it must be shown that the mediating variable changes before the outcome variable changes. However, the mediation model is grounded in theory. Research on the links between rumination and depression suggests that when someone is depressed they respond by continuously focusing on the reasons they might be depressed, their symptoms and what it means to them to be depressed [45]. The model is also supported by evidence suggesting that depressed mood alters information processing such that negative moods are more likely to result in negative evaluations and cognitions which leads people to think more negatively about the past [23, 45].

If people high in depression are thinking more negatively and evaluating life more negatively, it follows that they would think more about negative memories because these would come to mind more easily. Ergo, the more those memories are thought about, the less likely they are to fade. The model also raises the possibility of bi-directionality, that is, more negative thinking and less affect fade could in turn contribute to depression and increase or maintain it. For instance, greater depressive symptoms are linked with negative perceptual biases in which people underestimate how committed their romantic partners feel in their relationship, and in turn, this bias predicts the maintenance of depressed mood over subsequent days [46]. Future studies using, for example, a cross-lagged correlational design, could offer more evidence that the direction of the pathway is correct, and whether there is a reciprocal relationship.

An unforeseen limitation was the lack of specificity with which the question “how often have you written about this memory?” was asked. The scope was simply too broad, thus the writing question failed to measure what it intended to: does private writing offer therapeutic benefits and promote differential affect fade? Instead the question was left open to the participant to interpret and it is not clear what type of writing they were including or excluding when answering the question. Indeed, one person explicitly counted the recording of their memory for this study as a time in which they had written about their memory. It is expected that more traditional types of writing such as personal diaries were counted by participants but they likely included multiple types of writing that may better be classed as forms of communication and social contact. For example, in today’s society interpersonal communication involves myriad types of writing, from Facebook comments and messenger apps to texts and emails. In future studies the limitation of the writing question could be addressed by asking the question more specifically, perhaps referring to written forms of private reflection and giving examples of what participants should include and exclude. It may even transpire that outside of therapeutic settings, this kind of personal writing is uncommon.

### Conclusion

The goal of the current study was to examine how clinically-relevant levels of depressive symptomology and anxiety impact fading affect bias. The results show that at high levels of depressive symptomology, there was no evidence of differential affect fade—i.e., no FAB—and this relationship was mediated by the amount of time that one spent thinking about these negative autobiographical memories. Counter to our expectations, anxiety did not affect the occurrence of FAB in this study. The current paper is the first to offer evidence as to the mechanism (i.e., thinking about the event) that drives the link between depression and reduced affect fade.
